# Field Application of an Innovative Approach to Assess Honeybee Health and Nutritional Status

**DOI:** 10.3390/ani14152183

**Published:** 2024-07-26

**Authors:** Cecilia Rudelli, Roberta Galuppi, Riccardo Cabbri, Thomas Dalmonte, Luca Fontanesi, Giulia Andreani, Gloria Isani

**Affiliations:** 1 Department of Veterinary Medical Sciences, Alma Mater Studiorum-University of Bologna, Via Tolara di Sopra 50, Ozzano dell’Emilia, 40064 Bologna, Italy; cecilia.rudelli2@unibo.it (C.R.); roberta.galuppi@unibo.it (R.G.); rccabbri@gmail.com (R.C.); thomas.dalmonte2@unibo.it (T.D.); giulia.andreani2@unibo.it (G.A.); 2 Animal and Food Genomics Group, Division of Animal Sciences, Department of Agricultural and Food Sciences, Alma Mater Studiorum-University of Bologna, Viale Giuseppe Fanin 46, 40127 Bologna, Italy; luca.fontanesi@unibo.it

**Keywords:** honeybees, hemolymph proteins, vitellogenin, essential trace elements, *Varroa destructor*, *Nosema* spp.

## Abstract

**Simple Summary:**

Honeybees are vital pollinators, essential for maintaining ecosystems and biodiversity, but there is rising concern about the health of managed honey bee colonies, especially in heavily human-influenced ecosystems. Multiple factors contribute to this decline, including environmental conditions, forage quality, and pesticide use. These elements have a complex effect on the health and nutritional status of honeybee colonies and influence their response to disease and various stressors. In this study, the authors propose a new approach to assess colony health by correlating common measures of colony strength such as honey and pollen reserves, the number of bees, and brood with hemolymph proteins, common bee pathogens (*Varroa destructor* and *Nosema* spp.), and essential trace elements (iron, zinc, and copper). Significant correlations were found between hemolymph proteins and colony performance measures, and between *V. destructor* and hemolymph proteins and iron content. In conclusion, this study confirms the need for a more holistic approach to honeybee health considering all the relevant aspects and critical points that may affect colony survival.

**Abstract:**

Environment, forage quality, management practices, pathogens, and pesticides influence honeybee responses to stressors. This study proposes an innovative approach to assess colony health and performance using molecular diagnostic tools by correlating hemolymph proteins with common measures of colony strength, prevalent honeybee pathogens (*Varroa destructor* and *Nosema* spp.), and essential trace elements (iron, zinc and copper). Colonies were selected from four apiaries located in different environmental and foraging conditions in the province of Bologna (Italy). Hemolymph samples were taken from June to October 2019. The *Varroa* infestation of the colonies was estimated by assessing the natural mortality of the mites, while the bees were tested for *Nosema* spp. spores using a microscopic method. Hemolymph proteins were quantified and separated using SDS-PAGE, and colony performance was assessed by determining adult bees, total brood, honey, and pollen reserves. The biomarkers measured proved to be useful for monitoring changes in performance and trophic conditions during summer and early autumn. Significant correlations were found between hemolymph proteins and colony performance measures. A positive correlation between pollen reserves, vitellogenin, and hexamerin 70a highlights the importance of these proteins for successful overwintering. In October, *Varroa* infestation was negatively correlated with total proteins, vitellogenin, apolipophorin II, transferrin, and hexamerin 70a, with negative implications for overwintering; furthermore, *Varroa* infestation was also negatively correlated with iron content, potentially affecting iron homeostasis.

## 1. Introduction

Honeybees are one of the most important pollinating insects, providing an irreplaceable service in the maintenance and conservation of ecosystems and biodiversity. However, the complex relationship between honeybees and their environment is fraught with challenges that threaten their delicate balance. The loss of colonies poses serious problems for the integrity of ecosystems, particularly in areas where human intervention is greatest. In recent decades, the number of honeybee colonies has declined due to multiple causes [1]. Among these, environmental factors and forage quality play critical roles influencing the health and nutritional status of honeybee colonies [2] as well as the use of pesticides. These factors, in turn, affect the response of bees to disease and other stressors. Additionally, the season is an important factor, as honeybees, known for their adaptability, undergo profound physiological changes to meet the contrasting metabolic demands of summer and winter [3]. These changes include variations in hemolymph proteins and trace element contents in bees, which can be influenced by various factors such as malnutrition, disease, and exposure to pesticides. Trace element content, encompassing a range of essential chemical elements, is emerging as a key player in seasonal adaptation and disease response. For example, *Nosema*-infected bees have lower levels of iron, manganese, and nickel, which may contribute to their higher mortality during the overwintering period [4].

Among the bee pests, *Varroa destructor* and *Nosema ceranae* are the most widespread in Italy and currently the most difficult to control, both for the surviving honeybee and for the beekeepers. *V. destructor* (Mesostigmata: Varroidae) is a ubiquitous ectoparasitic mite known for its successful infestation of honeybee colonies worldwide. The *Varroa* mite is the leading cause of colony mortality in Europe, causing colony collapse and/or death in highly infested colonies if left untreated [5]. The second most common biological agents associated with worker bee decline are the obligate intracellular microsporidia of the genus *Nosema*, mainly *Nosema apis* and *Nosema ceranae*, both responsible for nosemosis in *Apis* spp. In particular, *N. ceranae* not only causes severe disease but also increases the susceptibility of honeybees to additional pathogenic infections by inducing immunosuppression [6,7,8]. At the colony level, *N. ceranae* infection is associated with a decrease in the size of the adult bee population, impaired brood rearing, reduced honey production, and an increased occurrence of queen replacement, contributing to increased colony losses [9,10,11,12]. Therefore, it would be interesting to have a tool such as hemolymph protein analysis to assess the nutritional and health status of honeybees in relation to the *V. destructor* and *Nosema* spp. infestation rates.

In this complex scenario, the traditional monitoring of colony welfare and performance, such as determining colony size, sealed brood, and stores, could be complemented by molecular diagnostic tools. All these variables should be closely monitored, especially during the productive season and the overwintering periods. In the spring and summer, it is crucial to analyze these variables to detect any early signs of crisis and thus mitigate the negative impacts on productivity and colony strength. Similarly, monitoring during the overwintering period is essential, as various factors—such as infections by *V. destructor*, *Nosema* spp., and inadequate nutrition—can exacerbate colony losses. It is therefore imperative to develop tools to improve the identification and understanding of the causes of these losses. In a previous study, the authors suggested that hemolymph total proteins, apolipophorin I and II, vitellogenin, transferrin, and hexamerin 70a may serve as a panel of biomarkers capable of assessing nutritional and health status at the colony level [13]. Considering these initial results, the aims of this research are to add other elements to clarify these associations by investigating the possible influence of the environment and the season on these proteins, searching for correlations between these proteins and commonly used measures of colony strength (brood, honey, and pollen stores), the prevalent honeybee pathogens (*V. destructor* and *Nosema* spp.), and three essential elements (iron, zinc, and copper).

## 2. Materials and Methods

### 2.1. Experimental Design and Colony Management

Four apiaries of *Apis mellifera ligustica* located in different sites of the province of Bologna (Italy) were selected. The first (identified as A) is located on the hill (San Martino in Pedriolo, municipality of Castel S. Pietro Terme). The environment is characterized by the presence of cereal crops, acacia trees, and wild grasses. The second (identified as B) is located in the plain area of Longara (municipality of Calderara di Reno) in an intensively cultivated landscape with the main crops being horticulture and vineyards, both cultivated using conventional methods. The third (identified as C) is an apiary subject to nomadism during the productive season. This apiary was initially set up in June in an agricultural setting in the locality of Gaiana (Municipality of Ozzano Emilia), mainly for the production of seed vegetables such as onions and carrots. It was then moved to Castel Guelfo, on seed alfalfa, and finally finished the season in Farneto (municipality of San Lazzaro di Savena), in a context characterized by abundant late-summer flowerings of ivy and inula. The fourth (identified as D) is located in Ponte Rizzoli (municipality of Ozzano Emilia) in a mixed landscape characterized by acacia, linden, and alfalfa.

For each apiary, 3 ten-frame Dadant–Blatt (DB) hives were randomly selected for the study (referred to as group A, group B, group C, and group D, respectively), for a total of 12 colonies. Samplings were carried out in June, July, August, September, and October 2019. Throughout this period, the beekeepers managed the colonies as usual and also administered *Varroa* treatments. In colonies from apiaries A, B, and D, brood blocking was performed on the 2nd, 14th, and 21st day of July, respectively, followed by oxalic acid treatment (Api-Bioxal^®^, Chemichals Laif, Padua, Italy) 25 days later. In contrast, the colonies in apiary C were treated against *Varroa* with Apitraz^®^ (Laboratorios Calier S.A., Barcelona, Spain) from 25 August to 18 October.

None of the colonies exhibited any clinical symptoms of disease during the experimental period.

### 2.2. Monitoring of Colony Performance

Colony performance and health status were checked monthly from June to October. During each visit, the colonies were monitored for functional control to assess the developmental status of the colonies. This control was carried out by determining various indices (total brood and adult population, amount of cells occupied by honey and pollen) according to the Liebefeld method, which was slightly modified [14,15,16]: during the inspection, each side of the frame was divided into six parts (later called “sixths”) and the number of sixths covered by each matrix was recorded. For each sixth, the empirical coefficients 250 and 750 were used to convert the area covered by bees or by cells containing brood, honey, or pollen, into the number of adult bees or cells present, respectively, according to Ugolini et al. [17].

### 2.3. Varroa Detection

The *Varroa* infestation in the colonies was estimated by assessing the natural fall of the mites [18], using adhesive diagnostic sheets. The sheets were changed three times every three days for a total of nine days of evaluation before each monthly visit. They were examined by counting each individual mite on the sheet, using a mechanical counter. The average daily fall and the total monthly fall were determined.

### 2.4. Bee Sampling

At each hive and during each sampling time, a minimum of 50 old worker bees were sampled from the outer frames (where foragers predominate). Additionally, 50 young worker bees were sampled between the last brood frame and the stores after a visual inspection of the frames to reduce age variability; typically, nursing bees are located on this frame. The bee samples, each contained in a jar with a mesh cap, were carefully transported alive to the laboratory of the Department of Veterinary Medical Sciences.

### 2.5. Nosema Detection

Thirty old worker bees from each colony were individually tested for *Nosema* spp. spores using microscopy (optical microscope Leica DMLS, Leica Microsystems Srl, Milan, Italy). The bees were anesthetized on ice, and their guts were extracted and placed in a 1.5 mL sterile Eppendorf tube containing 200 µL of deionized water. Each gut was then gently ground with a small pestle and 20 µL of the resulting suspension was examined under a microscope to assess the presence of *Nosema* spp. spores [11]. This method allowed for the detection of an expected 10% prevalence of infection, with 95% confidence in each group [19].

### 2.6. Hemolymph Sampling

From 30 young worker bees anesthetized in ice, 1–2 µL of transparent uncontaminated hemolymph was collected as previously described [13]. Hemolymph samples collected from bees of the same colony were pooled and stored at −80 °C.

### 2.7. Total Protein Determination

Total protein (TP) concentration was determined using the Bradford method (Bradford Reagent, Sigma-Aldrich, St. Louis, MO, USA). Bovine serum albumin (Sigma-Aldrich, St. Louis, MO, USA) served as the standard for the calibration curve. Absorbance was measured with a plate reader (Varioskan™ Lux, Thermo Fisher Scientific, Waltham, MA, USA).

### 2.8. Protein Separation Using SDS-PAGE

Hemolymph proteins were separated by 1D-SDS-PAGE electrophoresis. Diluted hemolymph was loaded onto 4–12% Bis-Tris polyacrylamide gels (NuPage/Thermo Fisher Scientific, Waltham, MA, USA), and electrophoresis was performed as described in [13]. Hemolymph samples were appropriately diluted to obtain 3 µg of total protein for loading onto the gel. Each gel also contained standard proteins of known molecular mass (SeeBlue™ Plus2 Pre-stained Protein Standard, Thermo Fisher Scientific, Waltham, MA, USA). Electrophoresis was performed at a constant voltage of 200 V for 40 min. Gels were stained with Coomassie G250. After staining, each gel was digitized using ChemiDocMP (Bio-Rad, Hercules, CA, USA), and pherograms were obtained with ImageLab 5.2.1 software (Bio-Rad, Hercules, CA, USA). The quantification of protein bands was performed as described in [13], using an internal standard of quantity (1 µg of protein) obtained by diluting 1:5 *v*:*v* a commercial solution of LDH (Sigma-Aldrich/Merck, Darmstadt, Germany) containing 5 mg of protein/mL. The volume of each protein band was determined by the software based on pixel density, and each volume was then compared to that of the internal standard of quantity, and the protein concentration was finally calculated. The identification of apolipophorin I and II, vitellogenin, transferrin, and hexamerin 70a in the bands was made based on the molecular mass and by comparison with the gels reported in [13].

### 2.9. Trace Element Determination Using Atomic Absorption Spectrometry

Trace elements were determined from the remaing young and old worker honeybees that were pooled into 10 pools for each time point after a careful washing of the bees by sequentially rinsing in two separate containers of deionized water. Honeybee pools (300 mg) were placed in individual acid-washed Teflon tubes and digested according to the method described by [20]. Briefly, samples were mixed with 1–2 mL of 65% HNO_3_ and 0.25–0.5 mL of 30% H_2_O_2_, digested in a microwave oven, transferred to 5–10 mL polyethylene volumetric flasks, diluted with bidistilled water, and analyzed using a flame atomic spectrophotometer (AAnalyst 100, PerkinElmer, Waltham, MA, USA). All the reagents were purchased from Merck (Darmstadt, Germany), with the acids and bidistilled water being of Suprapur grade. The accuracy of the method was assessed using a certified reference material (ERM^®^–BB422 fish muscle, Merck, Darmstadt, Germany). The concentrations found with the method used in this study fell within the certified uncertainty interval provided by ERM, corresponding to a 95% confidence level. The detection limits for flame atomic spectrophotometry were 0.09 µg/mL for Fe, 0.04 µg/mL for Zn, and 0.01 µg/mL for Cu. Trace element content was expressed as µg/g wet weight.

### 2.10. Statistical Analysis

Normality of distribution and equality of variances among groups were assessed using the Shapiro–Wilk and Levene tests, respectively [21,22]. Quade’s test and Quade’s all-pairs post hoc test were used to detect differences within the same groups among times [23,24]. Differences among groups at the same time were determined using Kruskal–Wallis and Dunn post hoc tests for multiple comparisons [23,25]. The correlation between values for each month was assessed using Spearman’s correlation [26]. A full dataset of *p*-values is reported in Supplementary Table S1. A *p*-value < 0.05 was considered statistically significant. Statistical analyses were performed using R 4.2.1 (R Foundation for Statistical Computing; Vienna, Austria; https://www.R-project.org/ accessed on 25 August 2023). Data are reported as mean ± SD (standard deviation).

## 3. Results and Discussion

### 3.1. Colony Performance

**Figure 1:**
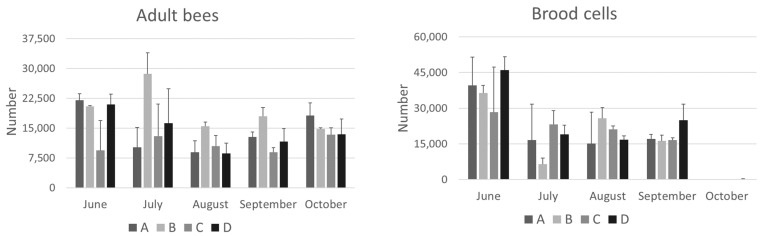
Adult bee population and brood cells measured at different time points from June to October in the hives of the four groups (A, B, C, and D) studied in the province of Bologna. The data are expressed as mean ± SD.

**Figure 2:**
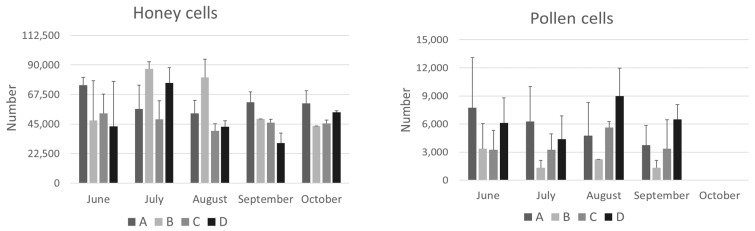
Cells of honey and pollen stores measured at different time points from June to October in the hives of the four groups (A, B, C, and D) studied in the province of Bologna. The data are expressed as mean ± SD.

The adult bee population, brood, honey, and pollen stores for each colony were estimated using the sixths method. These data are important for determining colony strength and are shown in Figure 1 and Figure 2.

In June, at the beginning of the study, the colonies considered in the different apiaries did not differ significantly from each other in terms of population consistency. Overall, the location of the apiary had no significant effect on the average bee population, with the exception of colonies from group B, which had a significantly higher population in September than those of group C (Figure 1).

When comparing brood, no significant differences were found between the colonies studied. However, a seasonal trend was observed over the months: in June, there was more brood, followed by a decrease in July to September, and a complete absence in October. This trend was consistent across all groups, including group C, where brood blocking was not performed. It is possible that this decrease in brood during the summer months is due to a reduction in pollen availability, which is more abundant in the spring, when honeybees primarily forage for nectar. The absence of brood in October is a physiological response to the beginning of the overwintering phase. A similar trend has been reported by other authors (e.g., [2]), who noted minimal brood from September to January.

On average, honey reserves did not differ significantly between the groups (Figure 2). The only exception was colonies from group A, which had significantly higher stores in September compared to groups B and D (*p* < 0.05). This difference could be attributed to the less anthropized environment of apiary A and the different foraging environment present there.

Pollen stores were not significantly affected by the season, except for their absence in October, which corresponds to the end of flowering. Regarding the influence of the site, on average there was a significant difference in August (*p* < 0.05): the pollen stores of the colonies in group B, located in an intensively cultivated landscape, were lower than those in groups C and D. In particular, higher pollen reserves were observed in September in group D (Figure 2). However, these reserves did not seem to affect the size of the colony population, or the amount of pollen stored until autumn. This suggests that the bees in site D likely consumed the pollen directly rather than storing it in the colonies. A similar trend in the late-summer–early-autumn period was also observed by Smart et al. [2].

### 3.2. Nosema and Varroa

It has been reported that the percentage of forager bees infected with *N. ceranae* is a useful indicator of the extent of disease in the colony [11]. Although we have not performed molecular analysis to identify the species involved in the present study, we have applied this index to *Nosema* ssp.

In our samples, there was a low prevalence of *Nosema* spp., ranging from 0 to 7% of infected foragers, with no significant differences between the apiaries. However, there were seasonal variations, with higher prevalence in June in all apiaries, and zero prevalence from July onwards, except in one colony in group B. This result is consistent with the findings of other authors. It is well known that the prevalence of *Nosema* spp. is highest in winter and spring, very low in the summer due to the increasing honeybee population, and starts to increase again at the end of autumn [27]. The seasonality of *N. apis* infection has long been recognized, whereas that of *N. ceranae* infection is still debated. Initial studies conducted in Spain showed no seasonal differences for *Nosema*, while a recent study performed in Germany showed a pattern essentially identical to that of *N. apis* [28].

Regarding the *Varroa* infestation, starting from a relatively consistent natural mortality in the different groups in June (ranging from 1 to 21 mites), various trends can be observed in July and August, corresponding to the periods of the treatments carried out in the different apiaries (Table 1). In group C, a higher average mortality was observed in September and October compared to the others, as it was still under treatment with antiparasitic strips. As previously observed, it should be noted that evaluating natural mortality is not always indicative of the actual infestation of the colony, as this is strongly influenced by the ratio of adult bees to brood [18]. In fact, in colonies of groups A and B, a higher extent of natural mortality was observed in October compared to June, which may be related to the lack of brood (Figure 1), leading to an increase in phoretic *Varroa* mites with a subsequent increase in natural mortality. On the other hand, in group D, a higher number of *Varroa* mortalities was observed in August, after treatment, indicating a higher infestation intensity; nevertheless, the natural mortality was lower in September and October when a higher presence of brood was observed with respect to the other colonies. This further emphasized the need for winter treatment to start the new season with a sufficiently low number of *Varroa* mites [29].

### 3.3. Hemolymph Proteins

**Figure 3:**
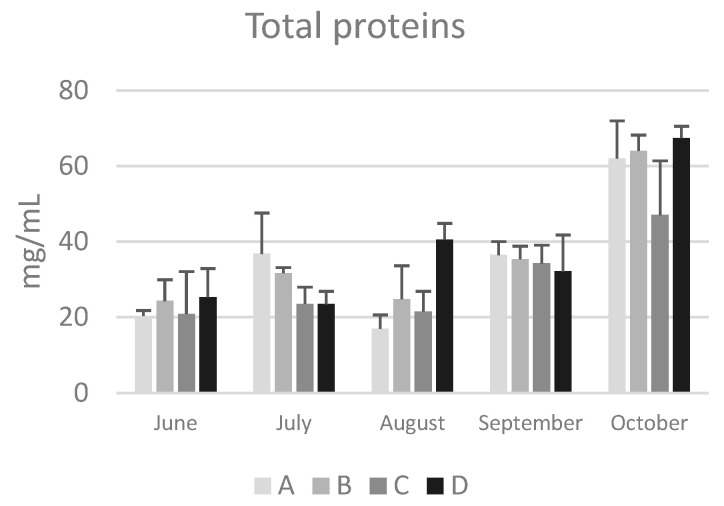
Total protein concentration in the hemolymph of honeybees sampled at different time points from June to October in hives of the four groups (A, B, C, and D) in the province of Bologna. Data are expressed in in mg/mL and reported as mean ± SD.

The location of the apiary site did not have a significant impact on the concentration of total protein, and the values remained consistent regardless of the environmental characteristics of the apiary areas (Figure 3).

As with seasonal variations, in June the concentration of total proteins in bee hemolymph varied from a minimum of 20.4 ± 1.4 mg/mL (group A) to a maximum of 25.3 ± 7.6 mg/mL (group D). By the end of October, the values ranged from a minimum of 47.0 ± 14.4 mg/mL (group C) to a maximum of 67.3 ± 3.2 mg/mL (group D), showing a significant increasing trend (*p* < 0.05) in all colonies except for those from group C. These values are consistent with previous reports by Cabbri et al. [30] and Isani et al. [13] in bees from the province of Bologna.

In agreement with previous studies [3,13,31], the data reported in this research confirm that long-living winter bees have higher concentrations of hemolymph total proteins compared to summer bees. Kunc et al. [3] proposed a physiological range of hemolymph total proteins for short-living and long-living bees in the Czech Republic, which can also be applied to honeybees from Emilia Romagna (Italy). The comparison shows a significant overlap of the intervals (Table 2), except for the upper limit in long-living bees, which is higher in winter bees from the Czech Republic. This could be due to total proteins continuing to increase during the winter, while in Italian bees sample collection ended in October (this study) or November [13].

**Figure 4:**
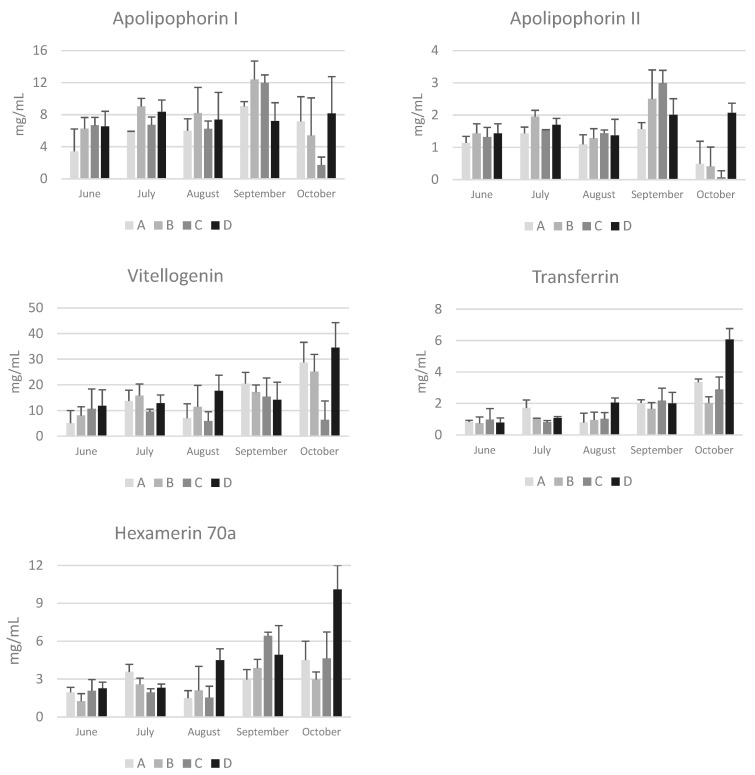
Concentrations of apolipophorin I and II, vitellogenin, transferrin, and hexamerin 70a in the hemolymph of honeybees sampled at different time points from June to October in hives of the four groups (A, B, C, and D) in the province of Bologna. Data are expressed in mg/mL and reported as mean ± SD.

Although post hoc tests did not reveal significant differences between groups due to the high variability among the colonies of the same group, in October the concentrations of apolipophorins, vitellogenin, transferrin, and hexamerin 70a were higher in bees from group D, particularly compared to those from group C, whose colonies were subject to nomadism (Figure 4). It is known that foraging environments with intensive agriculture negatively affect the nutritional status, the resistance to oxidative stress, and immune response [32], while areas characterized by a higher abundance of non-cultivated landscapes favor a higher success during the overwintering period. Smart et al. [33] reported that bees located in apiaries surrounded by greater floral abundance had higher levels of vitellogenin transcripts and experienced a higher survival rate during the winter than bees living in environments characterized by fewer blossoms and smaller foraging areas. Accordingly, Ricigliano et al. [32] reported that colonies in the proximity of less-cultivated environments showed elevated vitellogenin gene expression, suggesting an improved nutritional condition in these colonies compared to those living in more intensively cultivated lands. The data reported in the present study are particularly interesting due to the low concentration of vitellogenin and apolipophorin I and II (Figure 4) in October in colonies from group C. Despite an area characterized by abundant late-summer flowerings of ivy and inula, the colonies of this nomadic group have very low concentrations of these proteins. This suggests that the management practice is not optimal and leads to a poor nutritional status of the bees at the end of the production season. Similarly, a significant decrease in the lifespan of migratory adult bees compared to stationary bees has been reported by Simone-Finstrom et al. (2016) [34]. Regarding seasonal variations, an increasing trend from June to October was observed for vitellogenin, transferrin, and hexamerin 70a. This trend is in line with the increase in total proteins and confirms the finding from another study carried out in different apiaries of the Emilia Romagna region [13]. Due to variability among colonies within the same group, significant increases were only found in the colonies of group D for vitellogenin and transferrin (*p* < 0.01). Conversely, a significant decrease was observed in colonies of group C for apolipophorin I and II (*p* < 0.05). Apolipophorins are lipid transport proteins, and their concentration in the hemolymph is related to the lipid mobilization from the fat body [33]. Interestingly, the seasonal patterns of apolipophorin I and II differ from that of the other hemolymph proteins. These two lipid transport proteins did not show the increasing trend from June to October; notably, in October, the concentration of apolipophorin II was low in the colonies of groups A, B, and C, suggesting a reduced mobilization of lipids from the fat body to the tissues.

Finally, Kunc et al. [3] have proposed physiological intervals for vitellogenin in short-living and long-living bees in the Czech Republic. As for total proteins, physiological intervals can also be proposed for vitellogenin in short-living summer bees and long-living winter bees in Emilia Romagna (Table 3). Despite yearly variability, the data confirm a higher concentration of vitellogenin in winter bees, in accordance with the absence of brood.

### 3.4. Essential Trace Elements

Iron, zinc, and copper, the most abundant essential trace elements in living organisms, were measured in honeybees sampled in August, September, and October. The data on trace element content in honeybees are given in Table 4.

Significant differences between apiaries were found for iron and copper. In August, iron levels were significantly higher (*p* < 0.05) in the colonies of group A than in those of groups C and D. In October, significantly higher levels of iron and copper were found in the colonies of group B than in those of groups C and D (*p* < 0.05).

The seasonal influence was evident for iron and copper, with lower levels in winter bees, although the decrease from August to October was significant only for iron.

It is well known that the environment and the season influence the elemental content of honeybees, as reported in the literature [35,36]. However, the values found in this study are similar to those reported by Goretti et al. [35] (who found average values of 125.84 μg/g dry weight for iron, 132.47 μg/g dry weight for zinc, and 14.39 μg/g dry weight for copper in bees from apiaries in different locations in Umbria), Ćirić et al. [37] (who found values from 47.79 to 64.50 μg/g for iron, from 29.14 to 47.56 μg/g for zinc, and from 6.27 to 7.67 μg/g for copper in worker honeybees collected from different locations in Serbia), and Zarić et al. [36] (who reported intervals of 102 to 265 μg/g dry weight for iron, 60–142 μg/g dry weight for zinc, and 15–31 μg/g dry weight for copper in individual honeybees from Serbia and Austria), assuming a mean value of dry weight equal to 28% of wet weight [38].

In August, the great homogeneity of the values obtained, except for the iron value of 79.2 found in the colonies of group A, regardless of the location and the management of the apiaries, suggests that these values may represent the physiological levels of iron (between 46.7 and 49.2 µg/g), zinc (between 32.3 and 36.1 µg/g), and copper (between 8.41 and 8.89 µg/g) in healthy summer bees. For winter bees, there was a higher variability between groups, with values ranging from 23.3 to 38.5 μg/g wet weight for iron, from 28.2 to 41.5 μg/g wet weight for zinc, and from 5.7 to 10.6 μg/g wet weight for copper.

In winter bees, the level of zinc was higher than that of iron. Vitellogenin is the primary circulating zinc-carrying protein in honeybees. A recent study has shown that this protein can bind on average three Zn^2+^ ions/molecule [39]. As a consequence, the increase in zinc in winter bees may be related to the increase in vitellogenin. Due to their involvement in the immune defense system, this could be a physiological adaptation to cope with the cold season, with a higher potential to induce an immune response. The significant decrease in iron in winter bees may be related to several causes: reduced activity and opportunities to feed due to lack of flowering, and the need to reduce a possible source of oxidative stress to increase longevity. This finding is consistent with data reported by Ilijec et al. [40], while Ptaszyńska et al. [4] observed the highest iron content during winter in honeybees from Poland.

### 3.5. Correlations between Biochemical and Functional Parameters and Parasites

An analysis was conducted to determine possible correlations between biochemical, functional parameters, and parasites. The *p* values of Spearman correlation coefficients are reported in Supplementary Table S1.

In August, a significant negative correlation was found between total brood and pollen (*p* < 0.05), vitellogenin (*p* < 0.05), transferrin (*p* < 0.01), and hexamerin 70a (*p* < 0.05). It is logical that the more brood there is to feed, the fewer pollen stores there are. By October, there was no brood left (Figure 1), no pollen stores remaining (Figure 2), and the winter bees, whose hemolymph is rich in vitellogenin and hexamerin 70a, had become living protein stores.

The amount of pollen stores in June and August showed a significant positive correlation with total proteins (*p* < 0.05), transferrin (*p* < 0.01), and hexamerin 70a (*p* < 0.05). This correlation is particularly interesting in August, as the quantity and quality of pollen reserves accumulated during the late summer are crucial for successful overwintering. Similarly, Di Pasquale et al. [41] reported that vitellogenin and transferrin gene expression were significantly higher in bees fed with pollen than in bees that did not receive pollen. Furthermore, these authors also focused on the effects of different types of pollen, suggesting that not only the availability but also the quality of pollen are important for bee health.

In the present study, no correlation was found between the presence of *Nosema* spp. and biochemical or functional parameters. However, the prevalence of infected bees in the colonies studied was rather low and limited to the first observation period.

In the literature, it has been described that honeybees from colonies with low levels of *N. ceranae* infection have been found to produce more vitellogenin than bees from colonies with high levels, and this finding has been associated with colony resistance to *N. ceranae* [42]. Given that pollen diversity and nutritional quality can influence bee health, the diet may also influence the tolerance to *N. ceranae* [41,42,43]. In fact, healthy and *N. ceranae*-infected honeybees survive longer when they eat pollen, and the quality of the pollen strongly influences how the infection affects the bees [41].

Conversely, many significant correlations were found between the number of *Varroa* mites and hemolymph components. Honeybees usually overwinter with very low or no pollen reserves, as was the case with the colonies in this study (Figure 2). The hemolymph proteins of winter bees are crucial for survival and for brood nutrition in spring. In June, *Varroa* infestation was positively correlated with total proteins (*p* < 0.05) and transferrin (*p* < 0.05), while in October it was negatively correlated with total proteins (*p* < 0.05), vitellogenin (*p* < 0.01), apolipophorin II (*p* < 0.01), transferrin (*p* < 0.01), and hexamerin 70a (*p* < 0.01). This last finding suggests a reduced storage capacity and a reduced immune response in infested bees, mainly due to the lower concentration of vitellogenin, the most abundant protein in honeybee hemolymph, which is also involved in immunity. Accordingly, it has been reported that *Varroa* infestation affects the nutritional status of honeybees [44,45] and compromises their normal polyethism by altering the interaction between vitellogenin and juvenile hormone, two essential physiological components [46]. In addition, parasitization reduces the life expectancy and impairs immune system function, increasing the risk of viral infections. The negative correlation between *Varroa* infestation and vitellogenin is consistent with data previously reported by Kunc et al. [47]. These authors found that vitellogenin gene expression was significantly downregulated and hemolymph vitellogenin was lower in the *Varroa*-parasitized bees (1.19 ± 1.17 mg/mL) as compared to non-parasitized bees (7.75 ± 3.30 mg/mL). The negative effects of *Varroa* are due to the mite feeding on the fat body and hemolymph of immature and adult bees [48]. Low concentrations of total and specific hemolymph proteins during the critical pre-winter period may be a relevant factor in determining colony success or collapse after winter.

The homeostasis of essential elements is related to the health status of an organism. A close relationship between *Varroa* infestation and iron metabolism was evidenced by the negative correlation between them in August, suggesting a possible negative effect of the parasite on iron homeostasis. To the author’s knowledge, there are no data in the literature on the effect of *Varroa* on iron levels in honeybees, whereas *N. ceranae* infection has been found to cause iron deficiency in honeybees and to increase the production of transferrin, which binds and transports iron [49]. These findings suggest that *N. ceranae* may have developed a method of scavenging iron from the host, and that *N. ceranae* parasitism may lead to a significant reduction in total body iron reserves. Furthermore, Rodríguez-García et al. [49] reported that *N. ceranae* infection caused upregulation of the transferrin gene to counteract iron depletion by the pathogen, and Ptaszyńska et al. [4] suggested that the decrease in iron, manganese, nickel, and sodium observed in *Nosema*-infected bees compared to uninfected bees could be the reason for the higher mortality of *Nosema*-infected bees during the overwintering period.

Finally, Spearman correlation analysis also revealed many significant correlations among hemolymph proteins. From June to October, a significant positive correlation was observed between vitellogenin and total proteins, as well as between vitellogenin and transferrin and hexamerin 70a. This suggests an interplay between these multitasking hemolymph proteins related to brood nutrition and immunity.

## 4. Conclusions

In this study, the traditional assessment of colony welfare and performance was conducted by relating colony size, sealed brood, honey, and pollen stores, and the presence of *Nosema* and *Varroa* pathogens with molecular diagnostic parameters.

The location of the apiary and the surrounding environment are crucial factors for the success of a colony, as reported in numerous publications. Despite variations in locations and foraging environments, which can be considered representative of the landscape of the Emilia-Romagna region, the four groups selected for this study showed few significant differences. The management of the colonies appears to have a greater impact on determining the trophic state. The hemolymph proteins indicate that the colonies from group D are in a better trophic condition, whereas the colonies of group C, which are subject to nomadism, are in a poorer nutritional condition.

Biomarkers measured in honeybee hemolymph have proven to be useful for monitoring changes in trophic conditions during summer and early autumn. In October, the absence of brood and the high concentrations of total proteins in the hemolymph indicate the presence of winter bees. Since long-living bees are the ones that will survive the winter, their nutritional and health status are critical for the success of the colony. These bees have a high concentration of proteins, particularly vitellogenin, as well as transferrin and hexamerin 70a, which may play a key role in the physiology of winter bees.

Several interesting correlations were found between the biomarkers analyzed and the traditional performance indices. In particular, in June and August a significant positive correlation was observed between pollen stores and total proteins, transferrin, and hexamerin 70a, indicating a beneficial effect of pollen integration to prepare the colonies for the winter phase with sufficient reserves. On the other hand, in October, a negative correlation was observed between the *Varroa* infestation and the concentration of different hemolymph proteins, highlighting the importance of combating this parasite in the autumn and winter. In addition, a significant negative correlation between the *Varroa* infestation and the iron content was found, suggesting a detrimental effect of the parasite on iron metabolism also, with possible implications for immunity and orientation, which merits further studies in the future.

Finally, the data reported in this study confirm the abundance of vitellogenin, which is the major component of the hemolymph proteome, and consequently the importance of quantifying this protein as a nutritional biomarker at the colony level also. The proposed intervals for total proteins and vitellogenin could be considered as a first attempt to define physiological ranges in healthy summer and winter honeybees in Italy. In addition, other important proteins, including transferrin and hexamerin 70a, are certainly of great interest and deserve attention in future studies.

In a rapidly changing environment, a deeper understanding of the complexity of the relationships between honeybees, their environment, and beekeeping practices is essential. Many research questions remain to be answered. One of the major challenges is of how to conduct effective field monitoring of honeybee health and nutritional status. This study highlights the increasing need for comprehensive studies combining multiple methodological approaches to address this complex task.

## Figures and Tables

**Table 1:** Average monthly values of mite falls measured at different time points from June to October, measured in the hives of the four groups (A, B, C, and D) studied in the province of Bologna. The data are expressed as mean ± SD.

Month	Group A	Group B	Group C	Group D
June	7.67 ± 7.63	1.00 ± 1.41	3.33 ± 4.93	8.00 ± 11.12
July	729 ± 664	40.5 ± 57.3	831 ± 883	972 ± 1629
August	603 ± 873	454 ± 251	651 ± 621	2098 ± 3165
September	11.8 ± 5.56	46.6 ± 27.8	174 ± 198	19.1 ± 21.9
October	11.2 ± 3.18	20.7 ± 19.1	30.0 ± 25.2	3.60 ± 0.00

**Table 2:** Comparison of concentrations of hemolymph total proteins in short-living summer bees and long-living winter bees. Data are reported in mg/mL.

Short-Living	Long-Living	Reference
17.0–40.5 (July)	47.0–67.3 (October)	This study (min-max)
13.3–44.5 (July)	49.7–63.1 (November)	Isani et al. [13] (min-max)
12–42	49–87	Kunc et al. [3]

**Table 3:** Concentration of hemolymph vitellogenin in short-living summer bees and long-living winter bees. The data are reported in mg/mL.

Short-Living	Long-Living	Reference
9.54–15.8 (July)	6.40–34.6 (October)	This study (min-max)
5.70–12.5 (July)	23.4–41.1 (November)	Isani et al. [13] (min-max)
0–10	14–26	Kunc et al. [3]

**Table 4:** Trace element content in honeybees from four apiaries (A, B, C, and D) in the province of Bologna. The data are expressed in µg/g wet weight and reported as the mean ± SD.

	A	B	C	D
Iron				
August	79.2 ± 24.2 ^a,b,§^	49.2 ± 12.7	46.7 ± 9.40 ^a,§^	49.2 ± 7.70 ^b,§^
September	37.1 ± 12.9	50.6 ± 11.8 ^a^	29.6 ± 6.04 ^a^	36.8 ± 7.8
October	30.3 ± 7.37 ^§^	38.5 ± 5.30 ^a,b^	23.3 ± 1.99 ^a,§^	25.6 ± 5.17 ^b,§^
Zinc				
August	32.3 ± 10.9	32.8 ± 7.60	36.1 ± 9.55	34.55 ± 7.01
September	39.5 ± 17.2	40.0 ± 10.8	36.1 ± 9.55	30.5 ± 5.52
October	39.2 ± 10.4	41.5 ± 10.4	35.5 ± 6.95	28.2 ± 2.95
Copper				
August	8.58 ± 3.72	8.61 ± 1.70	8.41 ± 1.02	8.89 ± 1.74
September	9.12 ± 2.17	14.5 ± 2.61	8.64 ± 1.01	8.30 ± 1.14
October	8.64 ± 1.56	10.6 ± 2.07 ^a,b^	7.32 ± 1.87 ^a^	5.70 ± 0.56 ^b^

^a,b^ For each metal and within the same month, the same superscript in the same row indicates a significant difference between groups (*p* < 0.05). ^§^ For each metal and within the same group, the same superscript in the same column indicates a significant difference between time points (*p* < 0.05).

## Data Availability

All data reported in this study are available upon request.

## Supplementary Materials

The following supporting information can be downloaded at: https://www.mdpi.com/article/10.3390/ani14152183/s1, Table S1: *p*-values of Spearman correlation.
