# Supplementary material for: Field Application of an Innovative Approach to Assess Honeybee Health and Nutritional Status
Source: Animals (Basel). 2024 Jul 26;14(15):2183. doi: 10.3390/ani14152183 (PMC11311059; doi:10.3390/ani14152183)
Supplement: Supplementary file 1 [file animals-14-02183-s001.zip › animals-3076022-supplementary.pdf]

animals

MDPI

Supplementary Table S1: p values of Spearman correlation

Yellow box: significant correlations; in bold: negative significant correlations.

June

p-value	Bees	Var	Nos_prev	Nos_int	Nos_ab	Cov_tot	Honey	Pollen	Apo1	Vg	Apo2	Tf	Hex	PT
**Bees**		0,28422	0,13006	0,32473	0,46587	0,055576	0,3403	0,1983	0,098401	0,51089	0,21449	0,70093	0,65148	0,43954
**Var**	0,28422		0,94216	0,44246	0,80797	0,55913	0,1315	0,060846	0,40728	0,1012	0,72195	0,013475	0,12597	0,014234
**Nos_prev**	0,13006	0,94216		0,35409	0,12905	0,0769	0,76317	0,54649	0,033625	0,088202	0,12358	0,3636	0,12925	0,40721
**Nos_int**	0,32473	0,44246	0,35409		0,00023812	0,2104	0,88367	0,66742	0,94684	0,63826	0,72525	0,29147	0,89387	0,82535
**Nos_ab**	0,46587	0,80797	0,12905	0,00023812		0,6299	0,27695	0,47235	0,92032	0,97341	0,72525	0,80218	0,61407	0,96272
**Cov_tot**	0,055576	0,55913	0,0769	0,2104	0,6299		0,72928	0,97874	0,081553	0,12755	0,098401	0,089724	0,70093	0,36021
**Honey**	0,3403	0,1315	0,76317	0,88367	0,27695	0,72928		0,66814	0,46709	0,88104	0,80277	0,70093	0,93377	0,95749
**Pollen**	0,1983	0,060846	0,54649	0,66742	0,47235	0,97874	0,66814		0,80159	0,1468	0,50823	0,040874	0,0010044	0,012435
**Apo1**	0,098401	0,40728	0,033625	0,94684	0,92032	0,081553	0,46709	0,80159		0,00080667	0,001677	0,23454	0,32589	0,080974
**Vg**	0,51089	0,1012	0,088202	0,63826	0,97341	0,12755	0,88104	0,1468	0,00080667		0,038845	0,042646	0,038845	0,0053997
**Apo2**	0,21449	0,72195	0,12358	0,72525	0,72525	0,098401	0,80277	0,50823	0,001677	0,038845		0,59262	0,72928	0,5536
**Tf**	0,70093	0,013475	0,3636	0,29147	0,80218	0,089724	0,70093	0,040874	0,23454	0,042646	0,59262		0,0052995	0,0079996
**Hex**	0,65148	0,12597	0,12925	0,89387	0,61407	0,70093	0,93377	0,0010044	0,32589	0,038845	0,72928	0,0052995		0,080974
**PT**	0,43954	0,014234	0,40721	0,82535	0,96272	0,36021	0,95749	0,012435	0,080974	0,0053997	0,5536	0,0079996	0,080974	

July

p-value	Bees	Var	Nos_prev	Nos_int	Nos_ab	Cov_tot	Honey	Pollen	Apo1	Vg	Apo2	Tf	Hex	PT
**Bees**		0,59262	0,22287	0,22287	0,22287	0,48409	0,048167	0,082651	0,014552	0,37008	0,00061169	0,96819	0,93656	0,89443
**Var**	0,59262		0,76987	0,76987	0,76987	0,55545	0,65905	0,27164	0,61154	0,66958	0,29809	0,66886	0,63069	0,85254
**Nos_prev**	0,22287	0,76987		0	0	0,37008	0,22172	0,37008	0,11731	0,11731	0,11731	1	0,37008	0,55545
**Nos_int**	0,22287	0,76987	0		0	0,37008	0,22172	0,37008	0,11731	0,11731	0,11731	1	0,37008	0,55545
**Nos_ab**	0,22287	0,76987	0	0		0,37008	0,22172	0,37008	0,11731	0,11731	0,11731	1	0,37008	0,55545
**Cov_tot**	0,48409	0,55545	0,37008	0,37008	0,37008		0,052576	0,11731	0,66958	0,025984	0,93656	0,44062	0,41714	**0,025984**
**Honey**	0,048167	0,65905	0,22172	0,22172	0,22172	0,052576		0,42416	0,057254	0,17356	0,15907	0,54443	0,91528	0,48307
**Pollen**	0,082651	0,27164	0,37008	0,37008	0,37008	0,11731	0,42416		0,22287	0,45004	0,10974	0,48307	0,89443	0,57391
**Apo1**	0,014552	0,61154	0,11731	0,11731	0,11731	0,66958	0,057254	0,22287		0,63069	0,0013332	0,45739	0,40114	0,32589
**Vg**	0,37008	0,66958	0,11731	0,11731	0,11731	**0,025984**	0,17356	0,45004	0,63069		0,53722	0,02044	0,0044732	0,007282
**Apo2**	0,00061169	0,29809	0,11731	0,11731	0,11731	0,93656	0,15907	0,10974	0,0013332	0,53722		0,7388	0,87345	0,70921
**Tf**	0,96819	0,66886	1	1	1	0,44062	0,54443	0,48307	0,45739	0,02044	0,7388		0,0014273	0,078789
**Hex**	0,93656	0,63069	0,37008	0,37008	0,37008	0,41714	0,91528	0,89443	0,40114	0,0044732	0,87345	0,0014273		0,016471
**PT**	0,89443	0,85254	0,55545	0,55545	0,55545	**0,025984**	0,48307	0,57391	0,32589	0,007282	0,70921	0,078789	0,016471	

August

p-value	Bees	Var	Nos_prev	Nos_int	Nos_ab	Cov_tot	Honey	Pollen	Apo1	Vg	Apo2	Tf	Hex	PT	Fe	Zn	Cu
**Bees**		0,38546	0	0	0	0,10974	0,31182	0,080974	0,38546	0,87345	0,71861	0,3403	0,23454	0,81057	0,93487	0,096181	0,021776
**Var**	0,38546		0	0	0	0,83172	0,35503	0,50837	0,35503	0,66958	0,66886	0,91547	0,72928	0,60121	**0,045833**	0,069395	0,26746
**Nos_prev**	0	0		0	0	0	0	0	0	0	0	0	0	0	1	1	1
**Nos_int**	0	0	0		0	0	0	0	0	0	0	0	0	0	1	1	1
**Nos_ab**	0	0	0	0		0	0	0	0	0	0	0	0	0	1	1	1
**Cov_tot**	0,10974	0,83172	0	0	0		0,41714	**0,025069**	0,85254	0,055576	0,80022	**0,0062331**	**0,035287**	0,10514	0,75203	0,38938	0,50079
**Honey**	0,31182	0,35503	0	0	0	0,41714		0,077965	0,21155	0,53722	0,66886	0,38546	0,43344	0,56374	0,95583	0,66458	0,32684
**Pollen**	0,080974	0,50837	0	0	0	**0,025069**	0,077965		0,25036	0,13563	0,74273	0,0049657	0,027892	0,044801	0,76052	0,36329	0,34583
**Apo1**	0,38546	0,35503	0	0	0	0,85254	0,21155	0,25036		0,11731	0,0055746	0,55545	0,38546	0,27695	0,6191	0,79301	0,88199
**Vg**	0,87345	0,66958	0	0	0	**0,055576**	0,53722	0,13563	0,11731		0,29017	0,00080667	0,0037461	0,00049542	0,35987	0,66458	0,60062
**Apo2**	0,71861	0,66886	0	0	0	0,80022	0,66886	0,74273	0,0055746	0,29017		0,33189	0,16382	0,13993	0,53641	0,75203	0,86106
**Tf**	0,3403	0,91547	0	0	0	**0,0062331**	0,38546	0,0049657	0,55545	0,00080667	0,33189		0,00033017	4,5937E-05	0,26746	0,4813	0,40863
**Hex**	0,23454	0,72928	0	0	0	**0,035287**	0,43344	0,027892	0,38546	0,0037461	0,16382	0,00033017		0,00066307	0,46181	0,68395	0,31302
**PT**	0,81057	0,60121	0	0	0	0,10514	0,56374	0,044801	0,27695	0,00049542	0,13993	4,5937E-05	0,00066307		0,15466	0,71567	0,44752
**Fe**	0,93487	**0,045833**	1	1	1	0,75203	0,95583	0,76052	0,6191	0,35987	0,53641	0,26746	0,46181	0,15466		0,37195	0,67774
**Zn**	0,096181	0,069395	1	1	1	0,38938	0,66458	0,36329	0,79301	0,66458	0,75203	0,4813	0,68395	0,71567	0,37195		0,02139
**Cu**	**0,021776**	0,26746	1	1	1	0,50079	0,32684	0,34583	0,88199	0,60062	0,86106	0,40863	0,31302	0,44752	0,67774	0,02139	

September

p-value	Bees	Var	Nos_prev	Nos_int	Nos_ab	Cov_tot	Honey	Pollen	Apo1	Vg	Apo2	Tf	Hex	PT	Fe	Zn	Cu
**Bees**		0,9867	0	0	0	0,96	0,32509	0,48466	0,80218	0,4032	0,3932	0,34348	0,37356	0,52079	0,016647	0,21295	0,13416
**Var**	0,9867		0	0	0	0,24271	0,96012	0,26489	0,14966	0,34659	0,04254	0,92032	0,089724	0,98674	0,42504	0,82872	0,33421
**Nos_prev**	0	0		0	0	0	0	0	0	0	0	0	0	0	0	0	0
**Nos_int**	0	0	0		0	0	0	0	0	0	0	0	0	0	0	0	0
**Nos_ab**	0	0	0	0		0	0	0	0	0	0	0	0	0	0	0	0
**Cov_tot**	0,96	0,24271	0	0	0		0,068096	0,18904	0,13694	0,92032	0,63826	0,44296	0,70006	0,97341	0,84127	0,22018	0,22815
**Honey**	0,32509	0,96012	0	0	0	0,068096		0,1791	0,4444	0,45499	0,47649	0,38673	0,25842	0,89387	0,47649	0,46568	0,68573
**Pollen**	0,48466	0,26489	0	0	0	0,18904	0,1791		**0,023851**	0,77566	0,55384	0,89322	0,55384	0,56548	0,9068	0,80159	0,45605
**Apo1**	0,80218	0,14966	0	0	0	0,13694	0,4444	0,023851		0,31038	0,033041	0,24271	0,60322	0,42504	0,85481	0,4047	0,17735
**Vg**	0,4032	0,34659	0	0	0	0,92032	0,45499	0,77566	0,31038		0,80277	0,076538	0,82872	0,007547	0,98674	0,46709	0,94668
**Apo2**	0,3932	0,04254	0	0	0	0,63826	0,47649	0,55384	0,033041	0,80277		0,15408	0,04254	0,85481	0,34659	0,93377	0,48603
**Tf**	0,34348	0,92032	0	0	0	0,44296	0,38673	0,89322	0,24271	0,076538	0,15408		0,065646	0,080413	0,22018	0,53212	0,91317
**Hex**	0,37356	0,089724	0	0	0	0,70006	0,25842	0,55384	0,60322	0,82872	0,04254	0,065646		0,34659	0,073878	0,60322	0,93337
**PT**	0,52079	0,98674	0	0	0	0,97341	0,89387	0,56548	0,42504	0,007547	0,85481	0,080413	0,34659		0,70093	0,85481	0,69917
**Fe**	0,016647	0,42504	0	0	0	0,84127	0,47649	0,9068	0,85481	0,98674	0,34659	0,22018	0,073878	0,70093		0,17452	0,0024383
**Zn**	0,21295	0,82872	0	0	0	0,22018	0,46568	0,80159	0,4047	0,46709	0,93377	0,53212	0,60322	0,85481	0,17452		0,069717
**Cu**	0,13416	0,33421	0	0	0	0,22815	0,68573	0,45605	0,17735	0,94668	0,48603	0,91317	0,93337	0,69917	0,0024383	0,069717	

October

p-value	Bees	Var	Nos_prev	Nos_int	Nos_ab	Cov_tot	Honey	Pollen	Apo1	Vg	Apo2	Tf	Hex	PT	Fe	Zn	Cu
**Bees**		0,80038	0	0	0	0,63054	0,52822	0	0,033041	0,62719	0,81517	0,96024	0,88104	0,32823	0,60322	0,38484	0,073878
**Var**	0,80038		0	0	0	0,23779	0,24727	0	0,12211	**0,0063771**	**0,0033278**	**0,00035929**	**0,0096384**	**0,013928**	0,59867	0,25363	0,23794
**Nos_prev**	0	0		0	0	0	0	0	0	0	0	0	0	0	0	0	0
**Nos_int**	0	0	0		0	0	0	0	0	0	0	0	0	0	0	0	0
**Nos_ab**	0	0	0	0		0	0	0	0	0	0	0	0	0	0	0	0
**Cov_tot**	0,63054	0,23779	0	0	0		0,41012	0	0,87349	0,87349	0,24256	0,24413	0,1215	0,63054	0,63054	0,1215	0,41612
**Honey**	0,52822	0,24727	0	0	0	0,41012		0	0,10248	0,16797	0,19966	0,076992	0,056019	0,57481	0,98658	0,57481	0,59867
**Pollen**	0	0	0	0	0	0	0		0	0	0	0	0	0	0	0	0
**Apo1**	0,033041	0,12211	0	0	0	0,87349	0,10248	0		0,015801	0,20585	0,16152	0,32823	0,059972	0,51089	0,82872	0,67607
**Vg**	0,62719	**0,0063771**	0	0	0	0,87349	0,16797	0	0,015801		0,19889	0,033041	0,13833	0,0038149	0,67607	0,44584	0,93377
**Apo2**	**0,81517**	**0,0033278**	**0**	**0**	**0**	**0,24256**	**0,19966**	**0**	**0,20585**	**0,19889**		**0,0017911**	**0,009787**	**0,283**	**0,20585**	**0,26648**	**0,049846**
**Tf**	0,96024	**0,00035929**	0	0	0	0,24413	0,076992	0	0,16152	0,033041	0,0017911		0,00020447	0,033041	0,17394	0,13833	0,17394
**Hex**	0,88104	**0,0096384**	0	0	0	0,1215	0,056019	0	0,32823	0,13833	0,009787	0,00020447		0,089724	0,14966	0,11731	0,1869
**PT**	0,32823	**0,013928**	0	0	0	0,63054	0,57481	0	0,059972	0,0038149	0,283	0,033041	0,089724		0,96024	0,5334	0,62719
**Fe**	0,60322	0,59867	0	0	0	0,63054	0,98658	0	0,51089	0,67607	0,20585	0,17394	0,14966	0,96024		0,0051241	0,013906
**Zn**	0,38484	0,25363	0	0	0	0,1215	0,57481	0	0,82872	0,44584	0,26648	0,13833	0,11731	0,5334	0,0051241		0,047856
**Cu**	0,073878	0,23794	0	0	0	0,41612	0,59867	0	0,67607	0,93377	**0,049846**	0,17394	0,1869	0,62719	0,013906	0,047856	
